# Characteristics of auditory steady-state responses to different click frequencies in awake intact macaques

**DOI:** 10.1186/s12868-022-00741-9

**Published:** 2022-09-30

**Authors:** Tomoya Nakamura, Trong Ha Dinh, Makoto Asai, Hiroshi Nishimaru, Jumpei Matsumoto, Tsuyoshi Setogawa, Hiroyuki Ichijo, Sokichi Honda, Hiroshi Yamada, Takuma Mihara, Hisao Nishijo

**Affiliations:** 1 System Emotional Science, Faculty of Medicine, University of Toyama, Sugitani2630, Toyama, 930-0194 Japan; 2 Department of Anatomy, Faculty of Medicine, University of Toyama, Toyama, 930-0194 Japan; 3 Department of Physiology, Vietnam Military Medical University, Hanoi, 100000 Vietnam; 4 Candidate Discovery Science Labs, Drug Discovery Research, Astellas Pharma Inc., Tsukuba, Ibaraki 305-8585 Japan; 5 Research Center for Idling Brain Science (RCIBS), University of Toyama, Toyama, 930-0194 Japan

**Keywords:** Monkey, EEG, ASSR, ERSP, ITC

## Abstract

**Background:**

Auditory steady-state responses (ASSRs) are periodic evoked responses to constant periodic auditory stimuli, such as click trains, and are suggested to be associated with higher cognitive functions in humans. Since ASSRs are disturbed in human psychiatric disorders, recording ASSRs from awake intact macaques would be beneficial to translational research as well as an understanding of human brain function and its pathology. However, ASSR has not been reported in awake macaques.

**Results:**

Electroencephalograms (EEGs) were recorded from awake intact macaques, while click trains at 20–83.3 Hz were binaurally presented. EEGs were quantified based on event-related spectral perturbation (ERSP) and inter-trial coherence (ITC), and ASSRs were significantly demonstrated in terms of ERSP and ITC in awake intact macaques. A comparison of ASSRs among different click train frequencies indicated that ASSRs were maximal at 83.3 Hz. Furthermore, analyses of laterality indices of ASSRs showed that no laterality dominance of ASSRs was observed.

**Conclusions:**

The present results demonstrated ASSRs, comparable to those in humans, in awake intact macaques. However, there were some differences in ASSRs between macaques and humans: macaques showed maximal ASSR responses to click frequencies higher than 40 Hz that has been reported to elicit maximal responses in humans, and showed no dominant laterality of ASSRs under the electrode montage in this study compared with humans with right hemisphere dominance. The future ASSR studies using awake intact macaques should be aware of these differences, and possible factors, to which these differences were ascribed, are discussed.

**Supplementary Information:**

The online version contains supplementary material available at 10.1186/s12868-022-00741-9.

## Background

Auditory steady-state responses (ASSRs) are periodic evoked responses to various types of constant periodic auditory stimuli (e.g., click trains, amplitude-modulated tones, etc.). The functional integrity of the auditory neural system can be assessed by various parameters of ASSRs: examples of the parameters are power (e.g., event-related spectral perturbation [ERSP]) and a measure of the strength of phase-locked synchronization to specific events over all trials (e.g., inter-trial coherence [ITC]) ranging from 0 (non-phase-locked) to 1 (fully phase-locked over all trials) [1–3].

Previous cortical ASSR studies reported that ASSRs are maximal at stimulus rates of approximately 40 Hz in humans [2, 4] and that ASSRs dominate in the right hemisphere in humans (MEG: [5, 6]; EEG: [7–10]). These typical ASSRs patterns are disturbed in human psychiatric disorders, including schizophrenia, autism, and bipolar disorder [11–14]. These ASSR responses may reflect higher cognitive function; for example, the gamma band activity in ASSRs is impaired in cognitive disorders [15, 16] and is associated with the ability to temporarily store and manipulate information, which is required for pitch and speech perception skills [17–19].

One of the benefits of ASSR is that it can be similarly applied to animals: ASSR has been applied to rodents in various animal models of psychiatric diseases [20, 21]. On the other hand, non-human primates are important for translational research as well as an understanding of human brain function and its pathology, since non-human primates are closest to humans in terms of behavior and physiology as well as genetics [22–24]. However, ASSRs have not yet been reported in awake macaques. Furthermore, most electroencephalogram (EEGs) recording studies using awake monkeys were performed invasively by implanting electrodes to avoid motion noises (e.g., [25–28]). Recording of ASSRs from awake intact animals would be beneficial to translational research since it is consistent with animal ethics, especially in non-human primates [23], and easier and less time-consuming. We previously developed a system that allows EEG recordings of awake intact macaque monkeys during auditory stimulation [29]. In the present study, we analyzed ASSRs in awake intact macaques during the binaural presentation of click trains at 20 − 83.3 Hz.

## Methods

### Subjects

Five adult male rhesus macaques (*Macaca mulatta*) aged 9 − 13 years (body weight, 5 − 7 kg) were used. The subjects were housed in pairs in home cages with a 12-h-on/12-h-off light schedule with food (approximately 100 g/animal/day) and water available ad libitum. The cage size of the animals complied with the criteria for cage size for monkeys in the National Institute of Health Guide for the Care and Use Laboratory Animals, 8^th^ edition. Supplemental fruit and vegetables were provided daily. The weight of the animals was checked regularly, and their physical size and feces were checked daily by the experimenters and animal care staff under the guidance of a veterinarian. Environmental enrichment in the form of toys was performed daily. The subjects were treated in strict compliance with the Guidelines for the Care and Use of Nonhuman Primates in Neuroscience Research of the Japan Neuroscience Society and the Institutional Animal Care and Use Committee of Astellas Pharma Inc. This study was approved by the Institutional Animal Care and Use Committee of Astellas Pharma Inc. and accredited by the Association for Assessment and Accreditation of Laboratory Animal Care International (Permit Number: C-T12053, C-T12128, C-T13229, C-T14140, C-T15210, C-T15533, C-T17016, and C-T18027). All methods are reported in accordance with ARRIVE guidelines (https://arriveguidelines.org) for the reporting of animal experiments.

### Experimental setup

In this study, a noninvasive EEG recording system was used in awake macaque monkeys. Detailed information, instructions, and illustrations of this system have been previously reported [29]. Briefly, a thermoplastic net-like facial mask (Shell seat; Esform, Matsumoto, Japan) was molded to fit the face of each subject. The net-like facial mask was attached to a metal frame that was fixed to a monkey chair (O'HARA & Co., LTD., Tokyo, Japan).

All subjects habituated to the mask after training for 2 weeks. Briefly, a monkey sat in a monkey chair, and a face mask with a metal frame was fixed in the monkey chair. The mask held the anterior part of the head, whereas a U-shaped acrylic plate on the monkey chair held the posterior part of the head (i.e., the occipital bone below the inion). To present the auditory stimuli, two speakers were bilaterally placed 50 cm away from both sides of the chair.

### EEG recordings

**Fig. 1:**
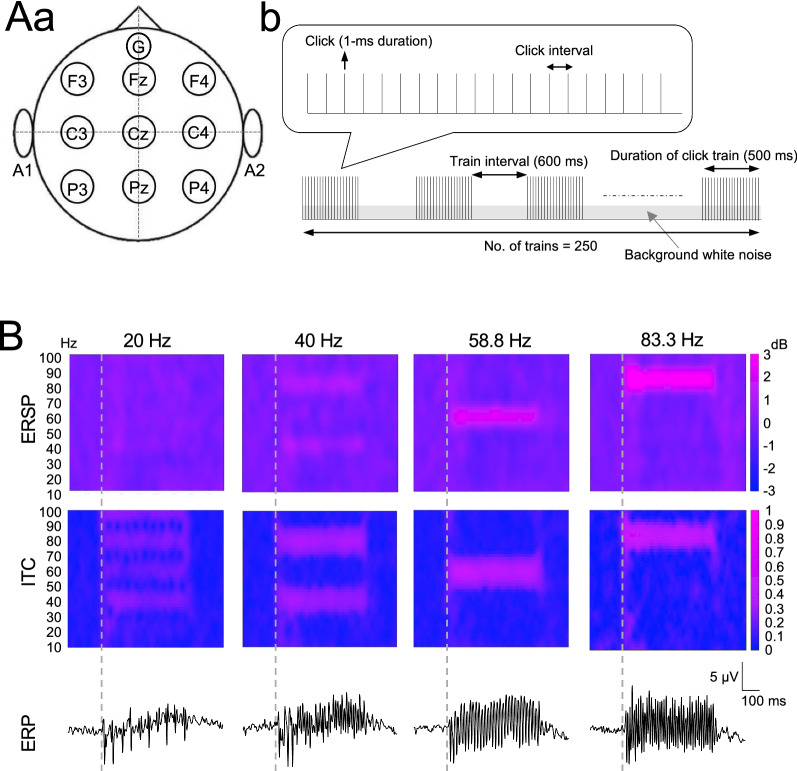
Mean auditory steady-state responses (ASSRs) in event-related spectral perturbations (ERSPs), inter-trial coherence (ITCs), and ERPs recorded from five subjects. **Aa** Placement of EEG electrodes. G, ground electrode. **Ab** Schematic diagram of 40-Hz click train. **B** Grand-averaged ASSRs at Cz in ERSPs (upper panel), ITCs (middle panel), and mean ERPs (lower panel) for five monkeys in response to click trains at 20, 40, 58.8, and 83.3 Hz. Dotted lines indicate stimulus onset. The y-axes indicate the spectral frequency of ERSPs and ITCs. Color bars indicate the values of ERSP (dB) and ITC (coherence) at each time-frequency point

The recording procedures were identical to those used in a previous study [29]. Briefly, the head of the subject was shaved in advance and fixed using a mask and monkey chair. A total of 11 active electrodes (F3, Fz, F4, C3, Cz, C4, P3, Pz, P4, A1, and A2) and a passive electrode for the ground (G) coated with Signa gel (Parker Laboratories, NJ, USA) were placed on the subject’s head according to the International 10–20 system and fixed by a surgical tape (Fig. 1Aa). The surface material of the electrodes was gold, and the diameter was 10 mm. EEG signals were recorded and amplified using a Polymate II AP216R2 system (Miyuki Giken Co., Ltd., Tokyo, Japan). All EEG channels were referred to the linked ear lobes for recording and analyses, and impedance was maintained below 30 kΩ. The EEG data were digitized at 1 kHz, and stored on a CF memory card.

### Auditory stimuli

According to previous studies that analyzed laterality index in humans [30, 31], two speakers were used in the present study. The 80 dB click train stimuli were presented to the subjects from two speakers under a 70 dB continuous background white noise generated by *sleep me* (Marpac, North Carolina, USA) (Fig. 1Ab). The intensity of the click train stimuli (80 dB) was determined according to the previous study with the same setup, in which clicks with this intensity elicited clear auditory evoked potentials in macaques [29]. In intact awake monkeys, EEG recording is susceptible to motion noises. Since clicks elicit larger ASSR responses than amplitude-modulated sounds [32], clicks instead of amplitude-modulated sounds were used in this study. Four types of click trains (83.3 Hz, 42 clicks/504 ms; 58.8 Hz, 30 clicks/510 ms; 40 Hz, 20 clicks/500 ms; 20 Hz, 10 clicks/500 ms) were generated by a sound generator (MT-ST-S, Melontechnos, Kanagawa, Japan), and presented in four separate blocks. For each click train frequency, 250 click trains were presented with a 590 − 600 ms interval.

### EEG data processing

EEG data were preprocessed using a digital Butterworth band-pass filter (12 dB/oct slope) from 0.5 to 100 Hz. The EEG data were segmented into 1000 ms epochs, including a 250 ms prestimulus interval. Specific epoch(s) in specific channel(s) of each subject were discarded if the peak magnitudes of the epochs exceeded the mean peak amplitudes of all epochs ± 2SD: average removal rate of 8.32 ± 0.13% (mean ± standard error of the mean [SEM]).

Analysis of EEG data was done using EEGLAB (Schwartz Center for Computational Neuroscience [33]): time-frequency analyses with short-term Fourier transformation were performed, and ERSP, power, and ITC were calculated. Power represents activity amount in specific frequency ranges of EEG signals based on Fourier transforms. ERSPs represent event-related changes in power relative to the prestimulus baseline, regardless of synchronization to clicks (power during the 250 ms prestimulus baseline was set at 0 dB in this study). ITCs represent phase-locked synchronization to clicks over all trials and range from 0 (non-phase-locked) to 1 (fully phase-locked over all trials). To estimate ASSRs in response to 20, 40, 58.8, and 83.3 Hz click trains, ITCs and ERSPs in the ranges of 18 − 22 Hz, 38 − 42 Hz, 56.8 − 60.8 Hz, and 81.3 − 85.3 Hz were computed and averaged during a 300 ms time window starting from 150 ms and ending at 450 ms after the onset of the first click, respectively. Powers of ASSRs in the ranges of 18–22 Hz, 38–42 Hz, 56.8–60.8 Hz, and 81.3–85.3 Hz were computed per epoch and averaged during the 300 ms time window starting from 150 ms and ending at 450 ms after the onset of the first click.

To examine left–right dominance in monkeys, the laterality index based on the mean powers and ITCs during the 300 ms time window starting from 150 ms and ending at 450 ms after the first click was computed at three AP levels (anterior: F4 vs. F3; middle: C4 vs. C3; posterior: P4 vs. P3). The mean value on the right side minus the mean value on the left side was divided by the sum of the right and left values [(R-L)/(R + L)]. It is noted that, according to the formulas for the laterality index, this index does not reflect right-left dominance in cases of negative values. However, ERSPs could be negative, since ERSPs are relative values between pre- and post-stimulation periods. In fact, some ERSP data showed negative values in this study. Therefore, instead of ERSPs, powers were used to compute laterality index.

Furthermore, differential potentials over the temporoparietal areas (anterior: F3-C3 and F4-C4; posterior: C3-P3 and C4-P4) were estimated to reduce the influence of the reference electrodes. We analyzed these data in the same way: we excluded epochs outside 2SDs of the data and computed powers and ITCs of ASSRs, as well as laterality indices using EEGLAB.

### Statistical analysis

Data are expressed as mean ± SEM. ERSPs, powers, and ITCs in the nine electrodes in response to the four types of click trains were analyzed using a repeated-measures two-way analysis of variance (ANOVA) with a mixed-effects model using restricted maximum likelihood (REML) followed by Tukey’s HSD test. In the two-way repeated-measures ANOVA REML mixed model, the denominator degrees of freedom in this analysis were adjusted using the Kackar-Harville correction [34, 35]. The data were weighted using the least-squares method [36]. This method (repeated-measures ANOVA REML mixed model) is less sensitive to small sample size bias and applicable to the fluctuating asymmetry model [37]. This method can reduce effects of variance inhomogeneity in repeated-measures ANOVA by the least squares method. However, it is possible that the data from the nine electrodes might not be independent, which might inflate statistical estimates, and that the statistical estimates could be affected by small sample bias (n = 5). To confirm the statistical results derived from the nine channels in two-way repeated-measures ANOVA REML mixed model (i.e., higher sensitivity to higher click train frequencies than to lower click train frequencies: see Results), we further analyzed the data derived from the one representative channel (Cz) with the bootstrap procedure. In this comparison, the ERSP and ITC values at Cz were resampled five times at each frequency by the bootstrap method using the data of the five animals, and the average value of the five resampled data was calculated. This resampling was repeated 2500 times at each click train frequency, and resultant data were compared among the four click train frequencies using the Kruskal–Wallis rank-sum test followed by the Steel–Dwass multiple comparison test.

Significant ASSR responses were defined based on coherency between the stimuli (clicks) and EEG signals, using ITC data. To analyze the coherency, mean ITC values during the 300 ms time window starting from 150 ms and ending at 450 ms after the stimulus onset were compared with those during 180 ms from −180 to 0 ms before the stimulus onset by two-way repeated-measures ANOVA REML mixed model [electrode × stimulus (pre-stimulus vs. post-stimulus period)] in each click frequency. The statistical results indicated a significant main effect of stimulus in all click frequencies (data not shown), indicating that EEG signals were more coherent to clicks after stimulus onset in all frequencies. Therefore, laterality dominance was analyzed in all click frequencies.

Data sets of laterality indices based on powers and ITCs were analyzed by a two-way repeated-measures ANOVA REML mixed model. To examine right-left dominance in detail, laterality index data were further analyzed using a bootstrap method. First, with a bootstrap resampling procedure using the powers and ITC data from the five monkeys, five powers and ITCs were generated on each side of each AP level, and the average value of the five data points was calculated for each side of each AP level. Using these resampled mean values, a laterality index based on powers and ITCs was computed for each AP level using a custom-written MATLAB script (MathWorks, MA, USA). This resampling was repeated 2500 times, and the laterality index was calculated 2500 times. Finally, it was estimated that the laterality index would show values greater than 0 or less than 0 (i.e., a significant deviation from 0 towards positive or negative values).

Statistical significance was set at *p* < 0.05. Statistical analyses were performed using JMP Pro 15.0 (SAS Institute, Cary, USA) and Excel (Microsoft, Redmond, USA).

## Results

### Representative data of ASSRs in ERPs, ERSPs, and ITCs

EEGs were recorded from nine electrodes placed on the monkey’s scalp (Fig. 1Aa). The four types of click trains (20 Hz, 10 clicks/500 ms; 40 Hz, 20 clicks/500 ms; 58.8 Hz, 30 clicks/510 ms; 83.3 Hz, 42 clicks/504 ms) were presented to record ASSRs (Fig. 1Ab). Grand averaged ERP waves for five monkeys evoked by repetitive click trains at 20, 40, 58.8, and 83.3 Hz are shown at the bottom of Fig. 1B. ASSRs in ERSP for five monkeys were observed around the same frequency band as the click frequencies when the click trains were presented at 40, 58.8, and 83.3 Hz, while ASSRs were not evident at 20 Hz (Fig. 1B). ASSRs in the ITC were also observed in response to click trains at 20, 40, 58.8, and 83.3 Hz (Fig. 1B).

### ASSRs in ERSPs and powers under different frequencies of click trains

**Fig. 2:**
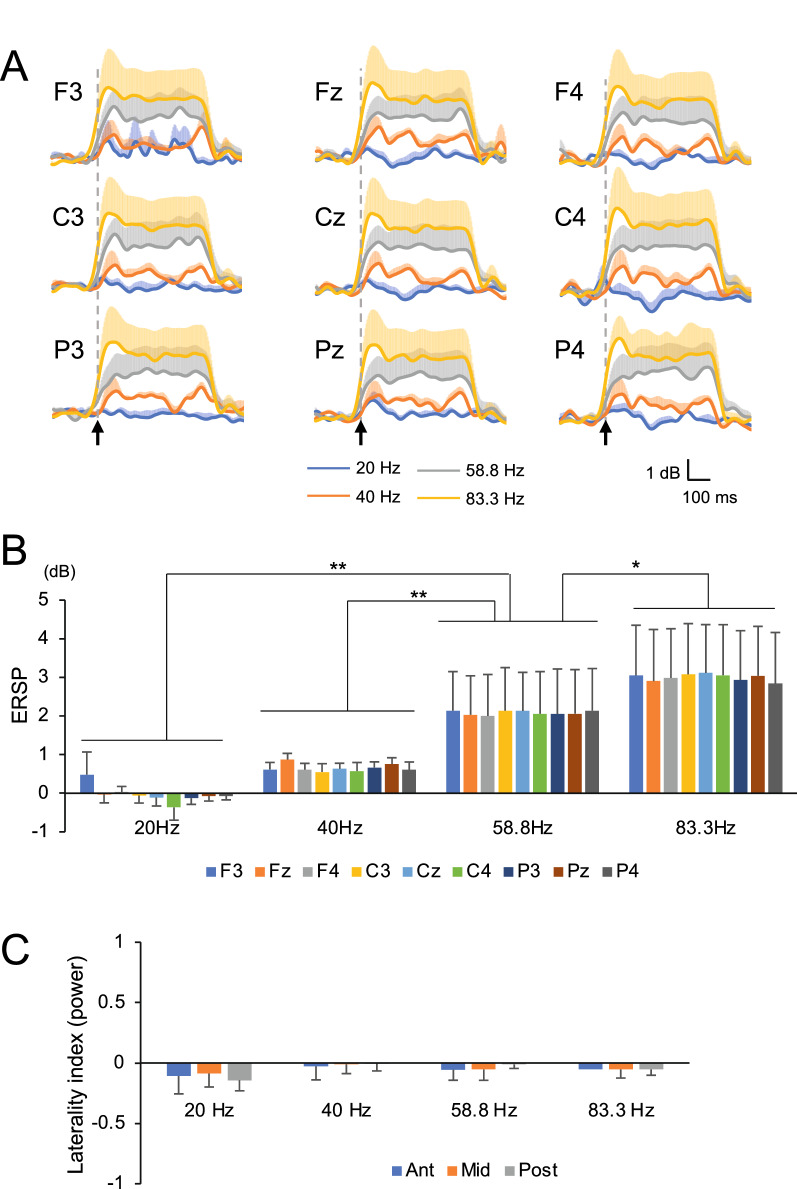
Comparison of the averaged ERSPs (**A**, **B**) and laterality indices of ASSRs (**C**) between different click train frequencies at 20 − 83.3 Hz. **A** Temporal changes in mean ERSPs across the five monkeys in response to 20, 40, 58.8, and 83.3 Hz click trains. Colored bars indicate SEM at each time point. **B** Comparison of mean ERSPs during 300 ms from 150 to 450 ms after stimulus onset for five monkeys in response to 20, 40, 58.8, and 83.3 Hz click trains at each electrode. **C** Comparison of mean laterality indices based on powers. Ant (blue), (F4-F3)/(F4 + F3); Mid (orange), (C4-C3)/(C4 + C3); Post (gray), (P4-P3)/(P4 + P3). An asterisk and double asterisks indicate significant differences (*p < 0.05, **p < 0.01, Tukey HSD test). Error bars represent SEMs

Figure 2A shows the temporal changes in the mean ERSP values of the five monkeys in response to 20, 40, 58.8, and 83.3 Hz click trains. In all electrodes, the ERSP values showed a plateau (stable state) during 300 ms from 150 to 450 ms after the first click in response to 40-, 58.8, and 83.3 Hz click trains, except for the 20 Hz stimuli. Therefore, to examine the effects of click train frequencies on ASSRs in ERSPs, we computed the mean ERSP during this period in each monkey in response to each click frequency. A statistical analysis of the mean ERSP values for 300 ms from 150 to 450 ms after the onset of the first click by two-way repeated-measures ANOVA REML mixed model (frequency × electrode) revealed a significant main effect of frequency (*F*_*3, 140*_ = 43.6097, *p* < 0.0001), but no significant main effect of electrode (*F*_*8, 140*_ = 0.0456, *p* = 1.00) or frequency × electrode interaction (*F*_*24, 140*_ = 0.0458, *p* = 1.00) (Fig. 2B) was observed. Post hoc comparisons indicated that the mean ERSP values were highest at 83.3 Hz compared to 58.8, 40, and 20 Hz (*p* = 0.0114, *p* < 0.0001, and *p* < 0.0001 for Tukey HSD test, respectively), and that these values were higher at 58.8 Hz than at 40 and 20 Hz (*p* < 0.0001 and *p* < 0.0001, respectively). These results indicated that ASSRs in ERSPs were more evident in higher frequency click trains up to 83.3 Hz in monkeys. We also analyzed the mean powers during the same period in the same way (Additional file 1: Fig. S1). A statistical analysis of the mean powers during 300 ms from 150 to 450 ms after the onset of the first click by two-way repeated-measures ANOVA REML mixed model (frequency × electrode) revealed a significant main effect of frequency (*F*_*3, 140*_ = 13.6527, *p* < 0.0001), but no significant main effect of electrode (*F*_*8, 140*_ = 0.0742, *p* = 0.9997) and no significant frequency × electrode interaction (*F*_*24, 140*_ = 0.0705, *p* = 1.00) were observed. Post hoc comparisons indicated that the mean power values were higher at 83.3 Hz than at 40 and 20 Hz (*p* = 0.0163 and *p* = 0.0003 for Tukey HSD test, respectively), and that these values were higher at 58.8 Hz than at 40 and 20 Hz (*p* < 0.0001 and *p* < 0.0001, respectively). These analyses indicated the results comparable to those in ERSPs: the powers in ASSRs were more evident at 58.8 and 83.3 Hz than at 20 and 40 Hz.

The above results (higher sensitivity to higher click train frequencies than to lower click train frequencies) could be ascribed to the small sample size (n = 5) or inflated statistical estimates due to simultaneous comparison of the data from the nine channels (see Methods). Therefore, we further analyzed the ERSP data derived from the one representative channel (Cz) with the bootstrap resampling procedure (Additional file 2: Fig. S2A). The results revealed a significant difference among the four click train frequencies (Kruskal–Wallis rank-sum test: χ^2^ = 8400.08, p < 0.0001). Post hoc comparisons indicated that the ERSP values were highest at 83.3 Hz compared to 58.8, 40, and 20 Hz (Steel–Dwass multiple comparison test: p < 0.0001 for all comparisons). Furthermore, ERSPs were higher at 58.8 Hz than at 40 and 20 Hz (Steel–Dwass multiple comparison test, p < 0.0001 for all comparisons), while ERSPs were higher at 40 Hz than at 20 Hz (Steel–Dwass multiple comparison test, p < 0.0001). Thus, the analysis of the single channel data of ERSPs indicated essentially comparable results to those of the nine channels.

Since ERSPs are relative values between pre- and post-stimulation periods, those values could be negative. Furthermore, since the formula of laterality index does not accept negative values, we analyzed laterality index using mean powers instead of ERSPs (see Methods). Figure 2C shows the laterality indices based on powers at three AP levels in response to 20, 40, 58.8, and 83.3 Hz click trains. To examine the effects of the frequencies of click trains on the laterality index, the laterality indices at three AP levels were analyzed using a repeated-measures two-way ANOVA REML mixed model (frequency × AP level). The statistical results indicated no significant main effects of frequency and AP level (frequency, *F*_*3, 44*_ = 2.0893, *p* = 0.1153; AP level, *F*_*2, 44*_ = 0.1238, *p* = 0.8838) and no significant frequency × AP level interaction (*F*_*6, 24*_ = 0.5908, *p* = 0.7358). These analyses indicated that no significant trends of laterality indices based on powers were observed in response to 20, 40, 58.8, and 83.3 Hz click trains.

To examine the right-left dominance of the laterality index based on powers, 2500 resampled data of the laterality index based on powers were generated at each AP level using the bootstrap resampling procedure. Examples of the resampled data of the laterality index in response to 83.3 Hz click trains are shown in Fig. S3A (Additional file 3). The results of the bootstrap tests indicated that the laterality index based on powers did not significantly deviate from 0 towards positive or negative values in each AP level: 20 Hz (anterior, *p* = 0.33; middle, *p* = 0.42; posterior, *p* = 0.37), 40 Hz (anterior, *p* = 0.48; middle, *p* = 0.50; posterior, *p* = 0.48), 58.8 Hz (anterior, *p* = 0.50; middle, *p* = 0.45; posterior, *p* = 0.48), and 83.3 Hz (anterior, *p* = 0.48; middle, *p* = 0.48; posterior, *p* = 0.48). Furthermore, laterality indices based on powers derived from differential potentials (anterior: F3-C3, F4-C4; posterior: C3-P3, C4-P4) were subjected to bootstrap resampling tests. The data in the same AP level were compared: F3-C3 vs. F4-C4 (anterior) and C3-P3 vs. C4-P4 (posterior). The results of bootstrap tests indicated that the laterality indices based on powers of differential potentials did not significantly (*p* > 0.05) deviate from 0 towards positive or negative values in each AP level in response to each click train frequency of 20 Hz (anterior, *p* = 0.32; posterior, *p* = 0.37), 40 Hz (anterior, *p* = 0.068; posterior, *p* = 0.30), 58.8 Hz (anterior, *p* = 0.068; posterior, *p* = 0.082), and 83.3 Hz (anterior, *p* = 0.25; posterior, *p* = 0.051). These analyses of powers derived from the bipolar recording indicated the results comparable to those in powers derived from the nine monopolar recordings: there was no significant left–right dominance in the monkey ASSRs in powers.

### ASSRs in ITC under different frequencies of click trains

**Fig. 3:**
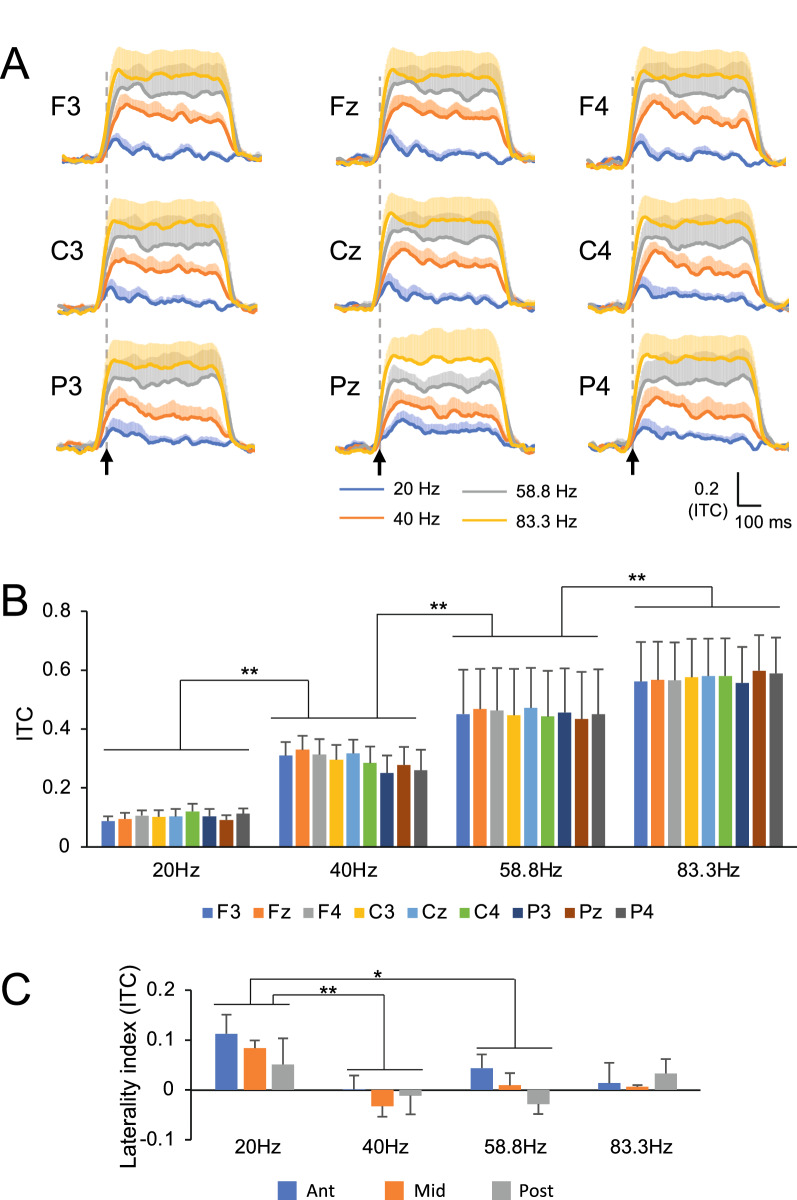
Comparison of the averaged ITCs (**A**, **B**) and laterality indices of ITCs (**C**) between different click train frequencies at 20 − 83.3 Hz. **A** Temporal changes in mean ITCs across the five monkeys in response to 20, 40, 58.8, and 83.3 Hz click trains. Error bars indicate SEM in each time point. **B** Comparison of mean ITCs during 300 ms from 150 to 450 ms after stimulus onset for five monkeys in response to 20, 40, 58.8, and 83.3-Hz click trains at each electrode. **C** Comparison of mean laterality indices based on ITCs. Ant (blue), (F4-F3)/(F4 + F3); Mid (orange), (C4-C3)/(C4 + C3); Post (gray), (P4-P3)/(P4 + P3). An asterisk and double asterisks indicate significant differences (*p < 0.05, **p < 0.01, Tukey HSD test). Error bars represent SEMs

Figure 3A shows the temporal changes in the mean ITC values across the five monkeys in response to 20, 40, 58.8, and 83.3 Hz click trains. In all electrodes, ITC values plateaued (steady state) during the 300 ms time window from starting 150 and ending 450 ms after the first click in response to 20, 40, 58.8, and 83.3 Hz click trains (Fig. 3A). Therefore, to examine the effects of the frequency of click trains on ASSRs in ITC (phase-locked synchronization with click trains), we computed the mean ITC during this period in each monkey in response to each click frequency. A statistical analysis of the mean ITC values during 300 ms from 150 to 450 ms after the first click using a two-way repeated-measures ANOVA REML mixed model (frequency × electrode) revealed a significant main effect of frequency (*F*_*3, 140*_ = 81,4215, *p* < 0.0001), but no significant main effect of electrode (*F*_*8, 140*_ = 0.0561, *p* = 0.9999) or frequency × electrode interaction (*F*_*24, 140*_ = 0.0655, *p* = 1.00) (Fig. 3B) was observed. Post hoc comparisons indicated that the mean ITC values were the highest at 83.3 Hz compared to those at 58.8, 40, and 20 Hz (*p* = 0.0013, *p* < 0.0001, and *p* < 0.0001 for Tukey HSD test, respectively); these values were higher at 58.8, 40, and 20 Hz (*p* < 0.0001 and *p* < 0.0001, respectively) and were higher at 40 Hz than at 20 Hz (*p* < 0.0001). These results indicate that ASSRs in ITC were more evident in higher click train frequencies up to 83.3 Hz in monkeys. To confirm the above results (i.e., higher sensitivity to higher click train frequencies), we further analyzed the ITC data derived from the representative single channel (Cz) with the bootstrap resampling procedure (Additional file 2: Fig. S2B). The results revealed a significant difference among the four click train frequencies (Kruskal–Wallis rank-sum test: χ^2^ = 8082.21, p < 0.0001). Post hoc comparisons indicated that the ITC values were highest at 83.3 Hz compared to 58.8, 40, and 20 Hz (Steel–Dwass multiple comparison test: p < 0.0001 for all comparisons). Furthermore, ITC values were higher at 58.8 Hz than at 40 and 20 Hz (Steel–Dwass multiple comparison test: p < 0.0001 for all comparisons), while those values were higher at 40 Hz than at 20 Hz (Steel–Dwass multiple comparison test, p < 0.0001). Thus, the single channel data analysis of ITCs indicated comparable results.

Figure 3C shows the laterality indices based on the ITCs at three AP levels in response to 20, 40, 58.8, and 83.3 Hz click trains. To examine the effects of click train frequency on the laterality index based on the ITC of monkeys, the laterality indices at three AP levels (anterior: F4 vs. F3; middle: C4 vs. C3; posterior: P4 vs. P3) were analyzed using a two-way repeated measures ANOVA REML mixed model (frequency × AP level). The statistical results revealed a significant main effect of frequency (*F*_*3, 44*_ = 5.1345, *p* = 0.0039), but no significant main effect of AP level (*F*_*2, 44*_ = 1.1257, *p* = 0.3336), and no significant frequency × AP level interaction (*F*_*6, 44*_ = 0.5251, *p* = 0.7862) (Fig. 3C). Post hoc comparisons indicated that the right dominant ITC laterality index was higher at 20 Hz than at 40 and 58.8 Hz (*p* = 0.0030 and *p* = 0.0309, respectively) (Fig. 3C). It is noted that the results indicated that the laterality index based on ITC was greater for 20 Hz click trains than for 40 and 58.8 Hz, which does not mean that the laterality index at 20 Hz deviated significantly from 0.

To examine the right-left dominance of the laterality index based on ITCs, 2500 resampled data from the laterality index based on ITCs were generated at each AP level using the bootstrap resampling procedure. Examples of the resampled data of the laterality index in response to 83.3 Hz click trains are shown in Fig. S3B (Additional file 3). We resampled the ITC data for all click frequencies in the same way, and the results of the bootstrap tests indicated that the distribution of resampled laterality indices based on ITCs did not significantly bias towards positive or negative values at each AP level in response to each click train frequency of 20 Hz (anterior, *p* = 0.17; middle, *p* = 0.27; posterior, *p* = 0.36), 40 Hz (anterior, *p* = 0.49; middle, *p* = 0.43; posterior, *p* = 0.45), 58.8 Hz (anterior, *p* = 0.47; middle, *p* = 0.50; posterior, *p* = 0.47), and 83.3 Hz (anterior, *p* = 0.47; middle, *p* = 0.49; posterior, *p* = 0.37). Furthermore, laterality indices based on ITCs derived from differential potentials (anterior: F3-C3, F4-C4; posterior: C3-P3, C4-P4) were subjected to bootstrap resampling tests. Data at the same AP level were compared: F3-C3 vs. F4-C4 (anterior) and C3-P3 vs. C4-P4 (posterior). The results of bootstrap tests indicated that the laterality indices based on ITCs of differential potentials did not significantly (*p* > 0.05) deviate from 0 towards positive or negative values in each AP level in response to each click train frequency of 20 Hz (anterior, *p* = 0.43; posterior, *p* = 0.23), 40 Hz (anterior, *p* = 0.102; posterior, *p* = 0.079), 58.8 Hz (anterior, *p* = 0.30; posterior, *p* = 0.47), and 83.3 Hz (anterior, *p* = 0.053; posterior, *p* = 0.76).

### Temporal changes in the ASSR-ITC laterality index

**Fig. 4:**
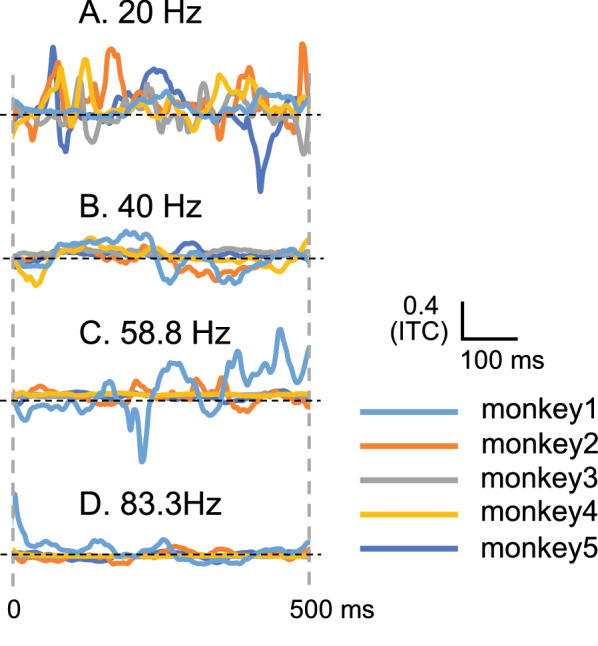
Instantaneous temporal changes in laterality indices of ITCs during presentation of the click trains in individual monkeys. **A**, **B**, **C**, and **D** indicate instantaneous temporal changes in laterality indices at 20, 40, 58.8, and 83.8 Hz of frequencies of click trains, respectively

The instantaneous laterality index was calculated for each monkey to investigate the temporal changes in the ITC laterality index (Fig. 4). In response to the 20 Hz click train, the polarity of the instantaneous laterality index (i.e., right-left dominance of ITC) was not stable; it changed over time, and the timing at which the right-left dominance of ITC switched differed between the subjects. Furthermore, the laterality index values were more stable but smaller in response to 40, 58.8, and 83.3 Hz click trains. These results showed that although the laterality index showed positive values in response to the 20 Hz click train (Fig. 3C), the left–right dominance of ITC at 20 Hz changed over time compared to that at other frequencies.

## Discussion

### Click train frequencies

In this study, ASSR responses based on ERSPs and ITCs were greater for the higher click train frequencies up to 83.3 Hz in macaque monkeys (Figs. 2A, B and 3A, B), in contrast to humans, in which ASSR responses are maximal at stimulus rates of approximately 40 Hz [2, 4, 38]. Consistent with the present results, a monkey neurophysiological study reported that multiple-unit activity in the auditory cortex showed phase-locked activity to click trains at click rates up to 150 Hz [39]. Two neural mechanisms have been proposed for the generation of ASSRs. One hypothesis is that ASSRs are formed by the superimposition of gamma-band responses at approximately 40 Hz, synchronized to each stimulus [40, 41]. In anesthetized macaque monkeys, gamma oscillations from 20 to 100 Hz, which include both phase-locked and non-phase-locked components, were elicited in the auditory cortex in response to tone bursts [42]. Interestingly, in this study, the oscillation frequency of the gamma bands gradually increased from 20 to 100 Hz during the initial 900 ms after stimulus onset [42]. These findings suggest that neurons in the auditory cortex might preferentially oscillate at higher gamma frequencies in macaque monkeys than in humans in a steady state. This might be the neurophysiological basis for the maximal response frequency of ASSRs at 83.3 Hz in this study, which is higher than in humans.

The second hypothesis for ASSR generation is that ASSRs are obtained by the superimposition of middle latency responses (MLRs) (series of waves that appear 8 − 80 ms after stimulus delivery) [38, 43]. Bohórquez and Özdamar experimentally tested this hypothesis using human subjects and reported that the superimposition of two biphasic waves (Na-Pa and Nb-Pb) of MLRs explained 93% of the ASSRs [43]. Furthermore, they reported that ASSR amplitudes derived from the superimposition of MLRs decreased at the 78 Hz stimulus repetition rate compared with those at 40 Hz, consistent with real ASSR data in humans. These findings suggest that the MLR waveform is an important factor in the generation of ASSRs and that the MLRs in macaque monkeys may be different from those in humans. Consistent with this idea, the peak latencies of MLR waveforms are shorter in macaque monkeys than in humans [43–45], suggesting that the spectral frequencies of MLR waves are higher in macaque monkeys than in humans. This finding further suggests that MLRs in macaque monkeys are more suitable for generating ASSRs at higher stimulus repetition rates than those in humans. These shorter latencies in MLRs in macaque monkeys might be attributed to a shorter temporal window of auditory integration, during which auditory information is combined and integrated, rather than the smaller brain sizes in macaques [45, 46]. An ASSR study in humans reported that ASSR amplitudes gradually increased from 40 ms to 200 − 250 ms after stimulus onset, suggesting that the temporal integration window for ASSR is approximately 200 ms [47]. In this study, ASSR amplitudes gradually increased up to 150 ms after stimulus onset (Fig. 2), suggesting that the temporal integration window may be shorter in macaque monkeys than in humans. Taken together, the results of this study (higher sensitivity to a higher stimulus repetition rate in macaque monkeys than in humans) may reflect the functional differences in auditory information processing between both species.

The above results (higher sensitivity to a higher stimulus repetition rate in macaques) might be ascribed to differences in sources of ASSRs between macaques and humans: ASSRs might more strongly reflect the activity of the subcortical sources of ASSRs in macaques while the activity of the cortical sources is dominant in human ASSRs. However, ASSR amplitudes are maximal at 40 Hz in the subcortical as well as cortical responses in humans [9, 48] although there is some individual variation in peak response frequencies ranging from 30 to 50 Hz in humans [49, 50]. These findings support the idea that the difference in the maximal frequencies between macaques (83.3 Hz) and humans (40 Hz) may be attributed to species differences rather than differences in the ASSR sources.

### Laterality of ASSRs

In this study, no significant laterality was observed in click train frequencies between 20–83.3 Hz under the electrode montage of this study in macaques (Figs. 2C, 3C, and 4). Some previous MEG studies reported left dominance at 20 Hz ASSR [51] and left dominance at 40 Hz ASSR only in left-handed females or no left-right dominance [52]. However, most studies reported that ASSRs were dominant in the right hemisphere in humans in various repetition rates and subjects (MEG: [5, 6]; EEG: [7–10]), in contrast with the present results in macaques. Several possible factors may explain the difference between macaques and humans (i.e., no ASSR laterality dominance in macaques compared with humans) (see below).

First, previous studies reported that sources of ASSRs or frequency-following responses (FFRs) were located in not only the cortical but also subcortical regions [2, 48, 53], while there is no laterality dominance in ASSRs recorded from the subcortical sources in various repetition rates [9]. The monkey cortex is about 17 times thinner than the human cortex [54], while the monkey skull is also thinner than the human skull [55, 56]. These findings suggest that the distance between the electrodes and subcortical areas is shorter in monkeys than in humans and consequently ASSRs might reflect more strongly subcortical activity in macaques. Therefore, ASSRs in this study could reflect activity in the subcortical sources, which may obscure laterality dominance.

Second, the electrode montage in the present study did not cover the whole cortical area: the lateral part of the head such as the temporal area was not recorded, which might also obscure laterality dominance. Although electrodes over the mastoid process could record signals from the temporal area in humans, the mastoid process is small and located on the bottom surface of the skull in macaques [57], which makes it difficult to place an electrode over the mastoid process in intact awake macaques.

Third, there are several morphological and physiological differences between macaques and humans. For example, the audible frequency range in macaques (55 Hz-45 kHz: [58, 59]) is higher than that in humans (20 Hz to 20 kHz: [60, 61]). However, the present study did not investigate ASSRs with click frequencies higher than 83.3 Hz. It is possible that higher frequency click trains might be more optimal for inducing ASSRs in macaques. The conclusion that there is no hemispherical difference only holds up to 83.3 Hz. Further studies with higher stimulus frequencies are required to confirm the absence of ASSR laterality in macaques.

Fourth, the absence of laterality dominance could be ascribed to linked-ear reference or volume conduction [62, 63]. In support of this idea, there were no significant differences among the nine electrodes in the present study, although previous human studies have reported that ASSRs were maximal in the vertex and/or middle frontal areas (e.g., [64, 65]). However, recent studies have reported that ASSR responses are distributed across a wider range of brain regions in humans, including the frontal, temporal, and parietal lobes, despite their varying strengths [10, 66, 67], which is consistent with our results. Human intracranial and scalp EEG recording as well as MEG studies reported that parietal and frontal cortical regions around the central sulcus are sensitive to periodic auditory stimuli including clicks with repetition frequencies of 20–100 Hz [9, 66–68]. The bipolar recording areas between F3-C3, C3-P3, F4-C4, and C4-P4 in the present study roughly correspond to these frontal and parietal regions, and there was no laterality dominance in these cortical areas. However, it is noted that more lateral regions of the hemispheres were not investigated in the present study (see above). Further studies are required to determine whether this finding of no laterality dominance holds true for the more lateral regions of the hemispheres in macaques.

## Conclusions

Non-human primates are important for translational research as well as an understanding of human brain function and its pathology, since non-human primates are closest to humans in terms of behavior, physiology, and genetics. In this study, to characterize ASSRs in non-human primates, we examined ASSRs in awake intact macaques (*Macaca mulatta*), while EEGs were recorded during presentation of click trains at 20 − 83.3 Hz. The present results based on ERSP and ITC demonstrated ASSRs, comparable to those in humans, in awake intact macaques. This study is the first to report robust ASSR responses in awake intact macaques. The ASSR recordings in monkeys should be useful as a translational tool since ASSRs are altered in various psychiatric diseases. It is interesting to investigate effects of chemicals such as ketamine and MK-801, which induces schizophrenia-like pathology [20, 21], on ASSRs in awake intact macaques. On the other hand, we found that there were some differences in ASSRs between macaques and humans: ASSRs were maximal at 83.3 Hz in macaques compared with humans with maximal frequencies at 40 Hz, while no laterality of ASSRs was observed in macaques compared with humans with right dominance. Future ASSR studies using awake intact macaques should be aware of these differences. Further studies are required to clarify the factors to which these differences are ascribed.

## Supplementary Information

**Additional file 1: Fig. S1.** Comparison of the averaged powers between different click train frequencies at 20-83.3 Hz. Mean powers during 300 ms from 150 to 450 ms after stimulus onset for five monkeys in response to 20, 40, 58.8, and 83.3-Hz click trains were computed at each electrode. **p < 0.01, *p < 0.05 (Tukey HSD test). Error bars represent SEMs.

**Additional file 2: Fig. S2.** Comparison of the ERSPs (A) and ITCs (B) at Cz among the four click train frequencies. The data were generated by bootstrap sampling. ****p < 0.0001 (Steel–Dwass multiple comparison test). Error bars represent SEMs.

**Additional file 3: Fig. S3.** Frequency distribution of laterality indices at 83.3 Hz of click train based on Powers (A) and ITCs (B) derived from bootstrap sampling. Ant, anterior; Mid, middle; Post, posterior.

## Data Availability

The datasets used and/or analyzed during the current study are available from the corresponding author upon reasonable request.
