# Supplementary material for: Characteristics of auditory steady-state responses to different click frequencies in awake intact macaques
Source: BMC Neurosci. 2022 Sep 30;23:57. doi: 10.1186/s12868-022-00741-9 (PMC9524006; doi:10.1186/s12868-022-00741-9)
Supplement: Supplementary file 1 — Additional file 1: Fig. S1. Comparison of the averaged powers between different click train frequencies at 20-83.3 Hz. Mean powers during 300 ms from 150 to 450 ms after stimulus onset for five monkeys in response to 20, 40, 58.8, and 83.3-Hz click trains were computed at each electrode. **p < 0.01, *p < 0.05 (Tukey HSD test). Error bars represent SEMs. [file 12868_2022_741_MOESM1_ESM.pdf]

Supplementary information

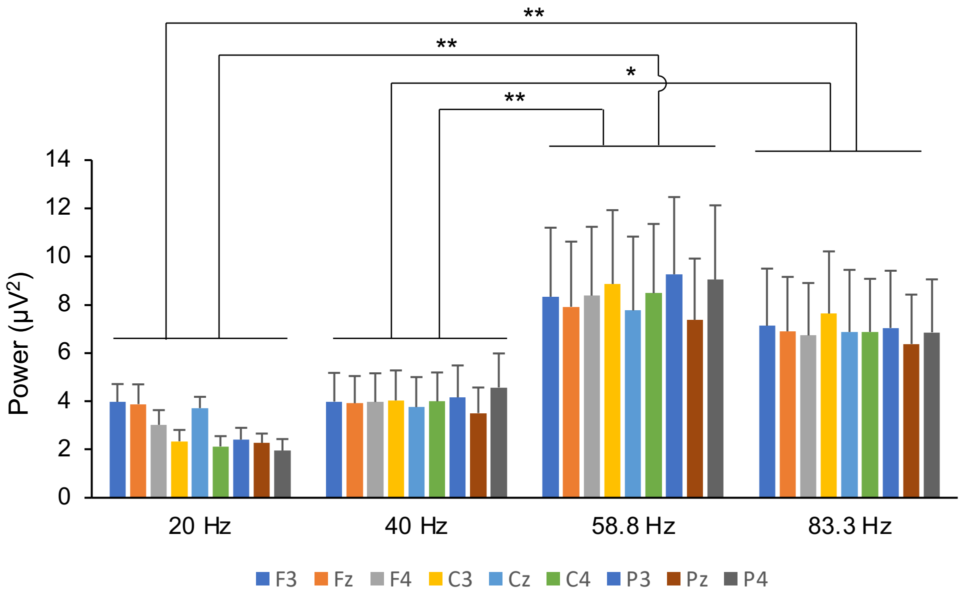

Additional file 1: Fig. S1. **Comparison of the averaged powers between different click train frequencies at 20-83.3 Hz.** Mean powers during 300 ms from 150 to 450 ms after stimulus onset for five monkeys in response to 20, 40, 58.8, and 83.3-Hz click trains were computed at each electrode. **$p < 0.01$, *$p < 0.05$ (Tukey HSD test). Error bars represent SEMs.
