# Supplementary material for: Characteristics of auditory steady-state responses to different click frequencies in awake intact macaques
Source: BMC Neurosci. 2022 Sep 30;23:57. doi: 10.1186/s12868-022-00741-9 (PMC9524006; doi:10.1186/s12868-022-00741-9)
Supplement: Supplementary file 2 — Additional file 2: Fig. S2. Comparison of the ERSPs (A) and ITCs (B) at Cz among the four click train frequencies. The data were generated by bootstrap sampling. ****p < 0.0001 (Steel–Dwass multiple comparison test). Error bars represent SEMs. [file 12868_2022_741_MOESM2_ESM.pdf]

Supplementary information

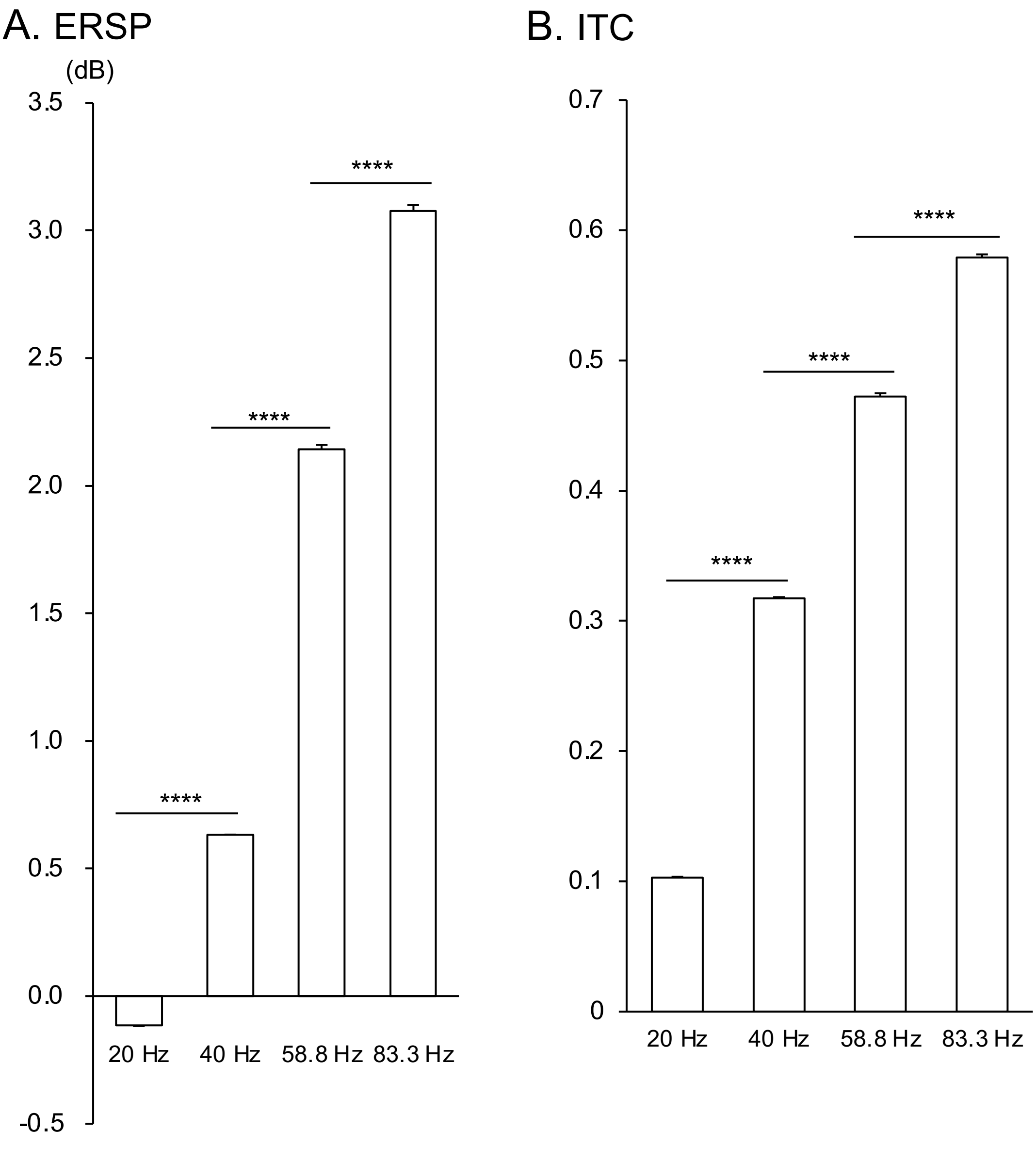

Additional file 2: Fig. S2. Comparison of the ERSPs (A) and ITCs (B) at Cz among the four click train frequencies. The data were generated by bootstrap sampling. ****$p < 0.0001$ (Steel–Dwass multiple comparison test). Error bars represent SEMs.
