# Supplementary material for: Characteristics of auditory steady-state responses to different click frequencies in awake intact macaques
Source: BMC Neurosci. 2022 Sep 30;23:57. doi: 10.1186/s12868-022-00741-9 (PMC9524006; doi:10.1186/s12868-022-00741-9)
Supplement: Supplementary file 3 — Additional file 3: Fig. S3. Frequency distribution of laterality indices at 83.3 Hz of click train based on Powers (A) and ITCs (B) derived from bootstrap sampling. Ant, anterior; Mid, middle; Post, posterior. [file 12868_2022_741_MOESM3_ESM.pdf]

Supplementary information

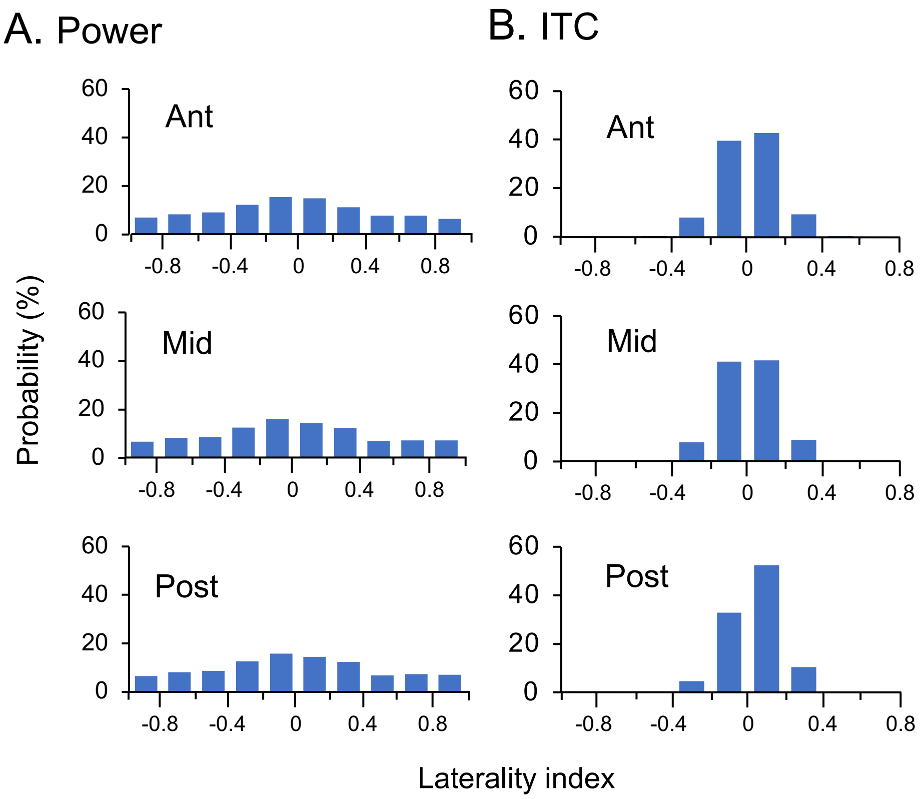

Additional file 3: Fig. S3. Frequency distribution of laterality indices at 83.3 Hz of click train based on Powers (A) and ITCs (B) derived from bootstrap sampling. Ant, anterior; Mid, middle; Post, posterior.
